# Flora of Northeast Asia

**DOI:** 10.3390/plants12122240

**Published:** 2023-06-07

**Authors:** Si-Qi Wang, Xue-Yun Dong, Liang Ye, Hong-Feng Wang, Ke-Ping Ma

**Affiliations:** 1 School of Forestry, Northeast Forestry University, Harbin 150040, China; bug_girl@live.com; 2 Northeast Asia Biodiversity Research Center, Harbin 150040, Chinaqingyege@gmail.com (L.Y.); 3 School of Geography and Tourism, Harbin University, Harbin 150040, China; 4 Folia Multidimensional Innovate Lab, Anshan 114000, China; 5 State Key Laboratory of Vegetation and Environmental Change, Institute of Botany, Chinese Academy of Sciences, Beijing 150093, China; kpma@ibcas.ac.cn

**Keywords:** diversity patterns, regional scale, mechanism, environmental drivers, hotspots

## Abstract

As a component of the MAP project, the study of the flora in Northeast Asia (comprising Japan, South Korea, North Korea, Northeast China, and Mongolia) convincingly underscores the indispensability of precise and comprehensive diversity data for flora research. Due to variations in the description of flora across different countries in Northeast Asia, it is essential to update our understanding of the region’s overall flora using the latest high-quality diversity data. This study employed the most recently published authoritative data from various countries to conduct a statistical analysis of 225 families, 1782 genera, and 10,514 native vascular species and infraspecific taxa in Northeast Asia. Furthermore, species distribution data were incorporated to delineate three gradients in the overall distribution pattern of plant diversity in Northeast Asia. Specifically, Japan (excluding Hokkaido) emerged as the most prolific hotspot for species, followed by the Korean Peninsula and the coastal areas of Northeast China as the second richest hotspots. Conversely, Hokkaido, inland Northeast China, and Mongolia constituted species barren spots. The formation of the diversity gradients is primarily attributed to the effects of latitude and continental gradients, with altitude and topographic factors within the gradients modulating the distribution of species.

## 1. Introduction

In the face of nature’s great diversity, classification is both an inherent need and an instinctive approach to make sense of it [1]. From plant taxonomy at the species level to vegetation classification on a global scale, flora can serve either as the outcome of such activities or as the material used for classifying the world around us [1,2,3,4]. More specifically, floristics is a discipline that studies the composition, distribution, and geographical elements of plants in a region based on various results of classification and discusses their origin and evolutionary history [5,6]. The theory and technology of phylogenetics have also been applied to the study of flora, which enriches its content [7,8,9]. This way, the floristic study includes the following aspects: comparing the similarity of different floras through composition analysis of plant assemblages at the level of family or genus; determining the geographical characteristics and origin of the flora based on a geographical elements analysis; and elucidating the phylogenetic composition of different geographical units, as well as their similarities, from an evolutionary perspective [5,9]. All these components largely contribute to our understanding of the flora in a certain region and its development over time.

Floristic research relies heavily on reliable biodiversity data, and in turn, comprehensive research can be used to enrich and improve the quality of these datasets. On the basis of species cataloging, it is of the first importance to study at length and fully understand the flora characteristics of each flora unit [4]. Asia as a whole is a data-poor region, the plant distribution data of Asia only accounts for 4% of the total on Global Biodiversity Information Facility (GBIF) [10]. As the accumulation of distribution data is essential for current biodiversity research, the Asian Biodiversity Conservation and Database Network (ABCDNet), administered by the Biodiversity Committee of the Chinese Academy of Sciences, launched the “Mapping Asia Plants (MAP)” project in November 2015 [11]. This project has the compilation of the database of plant checklists and distribution information as its core component [11]. The data are mainly collected through local floras, catalogs, atlases, specimen information, and field survey data, providing support for the completion of plant ecology, flora, plant geography, and other related research, enabling researchers to construct catalogs as well as gaining insight into the diversity and spatial structure of floras on a national or regional scale [11]. The MAP project divides Asia into six sub-regions: North Asia, Northeast Asia, Southeast Asia, Southwest Asia, South Asia, and Middle Asia [10,11].

**Figure 1:**
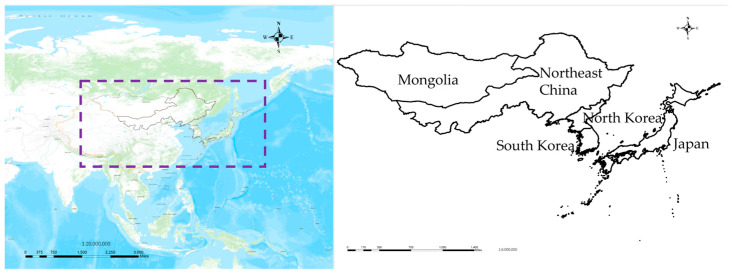
Northeast Asia, as illustrated in this Figure, comprises Japan, North Korea, South Korea, Northeast China (Heilongjiang, Jilin, Liaoning, and Inner Mongolia provinces), and Mongolia.

Northeast Asia, as illustrated in Figure 1, comprises Japan, the Korean Peninsula (North and South Korea), Northeast China (Heilongjiang, Jilin, Liaoning, and Inner Mongolia provinces), and Mongolia from east to west, covering a total area of approximately 4.1411 million km^2^, with a latitudinal range of 30° N to 49° N and longitudinal range of 88° E to 145° E [10,12]. Japan, being a long and narrow mountainous island country, features mostly a moist temperate monsoon climate, with Okinawa in the south having a subtropical climate and Hokkaido in the north a sub-frigid one [12,13,14]. The Korean Peninsula, exposed to the Sea of Japan on the east and the Yellow Sea on the west [15], is marked by the presence of high mountains in the northeast and low mountains or plains in the southwest [12,15]. Consequently, altitude is the main determinant of temperature and precipitation patterns [12]. With an area of 1.9703 million km^2^, Northeast China is composed of Heilongjiang, Jilin, Liaoning, and Inner Mongolia [15], where the Great Khingan Mountains are located in the north, the Little Khingan Mountains in the east, the Changbai Mountains in the south, and the Northeast Plain in the middle [16]. To the west, on the Mongolian Plateau where Inner Mongolia is bordered by the country of Mongolia, which is the second largest inland country in the world with a total 1.5665 million km^2^ landmass [15]. Finally, the climate of Northeast China and Mongolia is mainly typified by temperate continental monsoons, with the precipitation decreasing significantly towards the west [16].

Northeast Asia is characterized by a predominance of mountainous regions located in the contact zone between oceanic and continental geological platforms [17]. The climate of this area has remained stable since the Neozoic era and it was mostly unaffected by the Pleistocene glaciation [10,18]. It is widely accepted that the floristic composition present cover of Northeast Asia is mainly under the influence of two climatic gradients. The climate changes from temperate in the south to cold-temperate in the north and the continentality changes from the coast of the Pacific Ocean towards the interior [17,18]. According to Takhtajan’s system, Northeast Asia consists of seven floristic provinces: Altai-Sayan, Transbaikalia, Manchuria, Sakhalin-Hokkaidō, Japan-Korea, Volcano-Bonin, and Ryukyu [4]. The first two provinces of them belong to the Circumboreal region, the rest belongs to the East Asia region, and all seven belong to the Holarctic Kingdom [4].

The research on plants in Northeast Asia can be traced back as early as the 18th century [10]. From that time to the beginning of the 19th century, researchers from Britain, France, Germany, Austria, and especially Russia started the research, including the famous Carl J. Maximowicz of Russia, W. B. Hemsley of Britain and A. R. Franchet of France [10,19]. Afterward, Russian researchers took the lead in the floristic research of China, Japan, and Mongolia, and published “Primitiae Florae Amurensis” to list 985 species in Northern China and Mongolia [20], “Diagnoses breves plantarum novarum Japoniae et Mandchuriae” and “Diagnoses Plantarum Novarum Asiaticarum” to describe 1058 species in Northeast China and Japan [21,22]. By the beginning of the 20th century, Japanese researchers replaced Russian researchers and had a great impact on plant research in Northeast China and the Korean Peninsula. They published a series of books and catalogs that described over 2000 species in Northeast China and approximately 3000 species in the Korean peninsula, although some of them are not accepted today [10,23]. Since the middle of the 20th century, local researchers have started independent research on native plants and produced a large number of works with important influence represented by Floras, such as “Flora of China”, “Flora of Japan”, “Flora of Mongolia” and “The Genera of Vascular Plants of Korea”, published between 1995 and 2020 [10,11]. In 2017, “A Checklist of Woody Plants from Eastern Asia” was published, in which 152 families, 1264 genera, and 11,885 species of woody plants from China, Japan, and the Korean Peninsula were described [24]. It is one of the few monographs on plant species in Northeast Asia. Floristic research was performed locally and nationally from the late 20th century to the present, and no other monographs of flora have been presented on the Northeast Asia regional scale during this period [10].

The primary objective of this investigation is to describe and analyze the species and geographical compositions, as well as the fundamental status and attributes of threatened species, in the flora of Northeast Asia. The ultimate goal is to elucidate the distribution pattern of species diversity at a regional scale and the predominant factors influencing its formation. Providing comprehensive data and information on the flora of Northeast Asia can serve as valuable resources and references for identifying key areas for biodiversity conservation and informing policy decisions related to the conservation and management of natural resources. Simultaneously, delving into the traits and causes of species diversity patterns at regional scales can yield crucial insights into understanding the formation of plant diversity patterns at a broader scale.

## 2. Results

### 2.1. Floristic Composition of Floras in Northeast Asia

#### 2.1.1. Basic Composition of the Flora

There are a total of 225 families of native vascular plants, distributed in 1782 genera and 10,514 species (including infraspecific taxa) in Northeast Asia, of which ferns account for 7.70% of the total species, belonging to 17 families, 91 genera, and 810 species (including infraspecific taxa); lycophytes account for 0.52% of the total species, belonging to 3 families, 5 genera and 55 species (including infraspecific taxa); gymnosperms account for 0.87% of the total species, belonging to 7 families, 19 genera and 91 species (including infraspecific taxa); angiosperms accounts for 90.91% of the total species, belonging to 198 families, 1667 genera and 9558 species (including infraspecific taxa) (Table 1).

Among the 5 countries in Northeast Asia, Japan has the highest number of species and infraspecific taxa, totaling 6395, which is significantly higher than the numbers in the other 4 regions: North Korea (2119), South Korea (2463), Mongolia (2920), and Northeast China (3810). In terms of specific plant species, the number of gymnosperms in Northeast China is the largest, at 45. Japan demonstrates superiority in terms of the number of ferns and lycophytes, with a total of 807, far higher than that in the other four regions. Notably, South Korea also has a quite high number of ferns and lycophytes, totaling 244. Correspondingly the proportion of angiosperms in the total number of plants is relatively low in that country (89.32%), followed by Japan (86.69%). Conversely, the proportion of angiosperms is the highest in Mongolia (97.71%) and is relatively high in Northeast China (95.33%) and North Korea (93.16%).

#### 2.1.2. Analysis of the Components of the Families

Based on the species (including infraspecific taxa) richness of families, 5 family levels have been classified: large families containing more than or equal to 100 species; secondary large families containing from 50 to 99 species; medium families containing from 10 to 49 species; small families containing from 2 to 9 species; and single-species families containing only one species.

Among the 225 families recognized in Northeast Asia, 24 large families have particularly high species and infraspecific taxa richness, representing 68.55% of the total species. These families are Asteraceae (1133), Poaceae (706), Cyperaceae (643), Fabaceae (585), Rosaceae (439), Ranunculaceae (390), Lamiaceae (310), Aspleniaceae (305), Polypodiaceae (292), Orchidaceae (291), Brassicaceae (241), Caryophyllaceae (215), Apiaceae (209), Ericaceae (187), Polygonaceae (166), Amaranthaceae (145), Orobanchaceae (142), Salicaceae (131), Plantaginaceae (123), Boraginaceae (115), Rubiaceae (115), Violaceae (114), Saxifragaceae (112), Caprifoliaceae (100).

**Figure 2:**
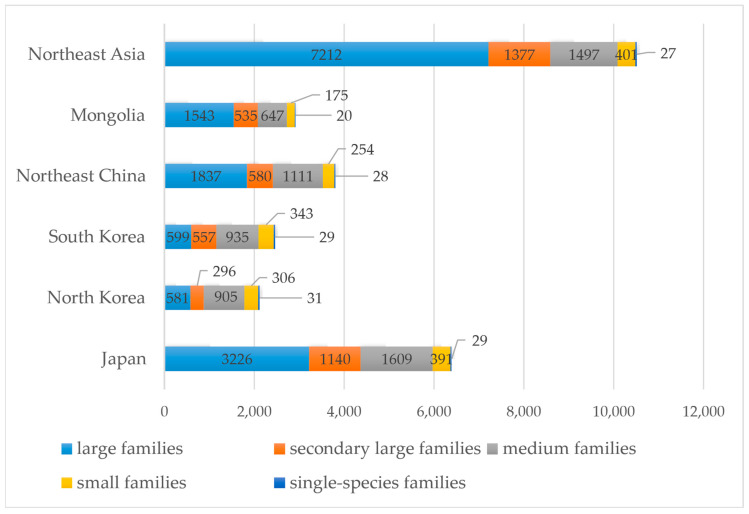
Distribution of 5 family levels in the flora of Northeast Asia and the other 5 regions. Large families: ≥100 species and infraspecific taxa; secondary large families: 50–99 species and infraspecific taxa; medium families: 10–49 species and infraspecific taxa; small families: 2–9 species and infraspecific taxa; single-species families: 1 species or 1 infraspecific taxon. The numbers on the bar indicate the total number of species and infraspecific taxa included at the family level.

As in Northeast Asia, large families are the most contributing in Mongolia, Northeast China, and Japan, accounting for 52.84%, 48.16%, and 50.45% of the total, respectively (Figure 2). In Mongolia, 7 large families are Asteraceae (435), Fabaceae (327), Poaceae (224), Brassicaceae (158), Rosaceae (148), Ranunculaceae (129), and Cyperaceae (122). In Northeast China, 8 large families are Asteraceae (456), Poaceae (309), Cyperaceae (255), Fabaceae (215), Ranunculaceae (190), Rosaceae (173), Caryophyllaceae (121), and Lamiaceae (118). Additionally, in Japan 12 large families are Asteraceae (542), Cyperaceae (451), Poaceae (418), Polypodiaceae (281), Aspleniaceae (280), Orchidaceae (260), Rosaceae (221), Fabaceae (192), Ranunculaceae (169), Ericaceae (160), Lamiaceae (152), and Apiaceae (100). 

In contrast, in North Korea and South Korea, the medium families contribute the most species and infraspecific taxa, accounting for 42.67% and 37.93% of the total, respectively. The number of large families is relatively small, with only three in North Korea and South Korea, accounting for as low as 27.49% and 24.38% of the total, respectively. They are Cyperaceae (233), Asteraceae (188), and Poaceae (160) in North Korea while Cyperaceae (245), Asteraceae (189), and Poaceae (165) in South Korea.

**Figure 3:**
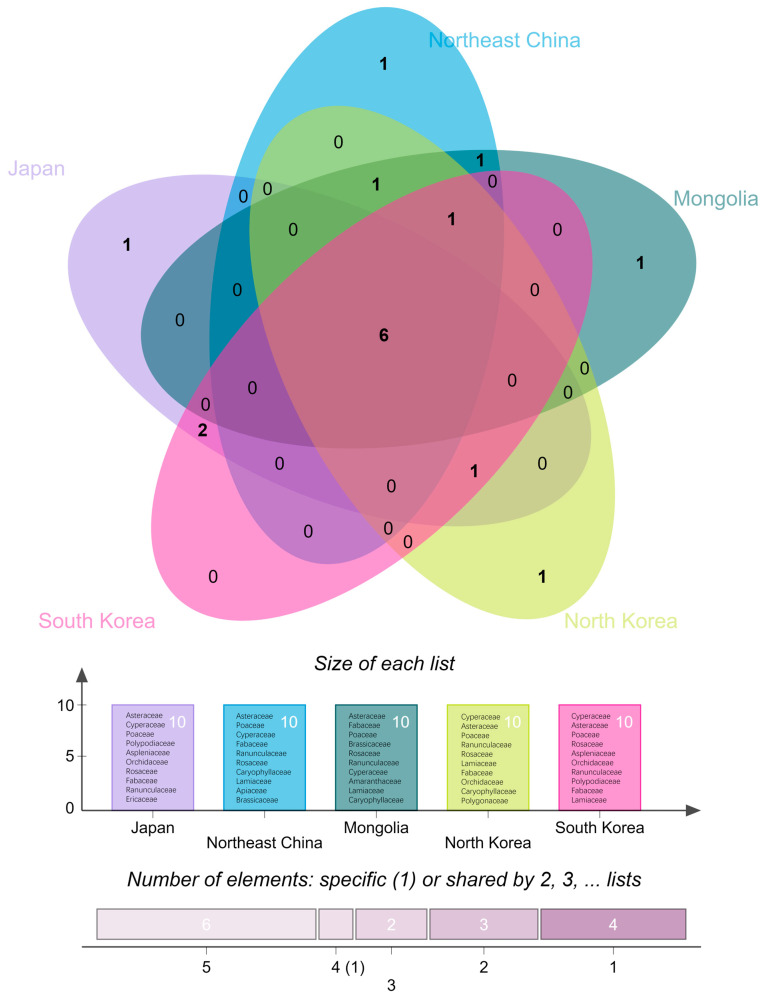
Similarity analysis of the dominant families in five regions: Japan, Northeast China, North Korea, South Korea, and Mongolia. The size of each list represents the number of dominant families, and the number of elements indicates the number of dominant families that are either dominant to only one region or shared by two, three, four, or five regions. The number at each intersection shows the number of shared dominant families.

Dominant family (genus) refers to the family (genus) which contains relatively more species and is typical taxa of particular regions. The main body of the flora of a region usually consists of dominant families (genera), so these families (genera) play important roles in the species origin and floristic research of the region. In this paper, we have defined the top ten largest families as the dominant families in each region, given that they all contribute about half of the species and infraspecific taxa in their respective regions. In terms of the similarity of the dominant families in these five regions, there are six dominant families shared by Japan, North Korea, South Korea, Northeast China, and Mongolia; they are Asteraceae, Cyperaceae, Poaceae, Rosaceae, Fabaceae, Ranunculaceae. Lamiaceae is shared by four other regions except Japan. The common dominant families in the three regions are as follows: North Korea, Northeast China, and Mongolia share Caryophyllaceae; Japan, North Korea, and South Korea share Orchidaceae. Three dominant families are shared by both countries, and Polypodiaceae and Aspleniaceae are shared by Japan and South Korea; Brassicaceae are shared by Northeast China and Mongolia. Ericaceae are the dominant family in Japan but not in other regions, Apiaceae are the dominant family in Northeast China while not in other areas, Amaranthaceae are the dominant family in Mongolia but not in other regions; Polygonaceae are the dominant family in North Korea but not in the other regions. The dominant families in South Korea are also dominant families in other regions, and South Korea does not have any unique dominant families that differ from other areas (Figure 3). 

**Figure 4:**
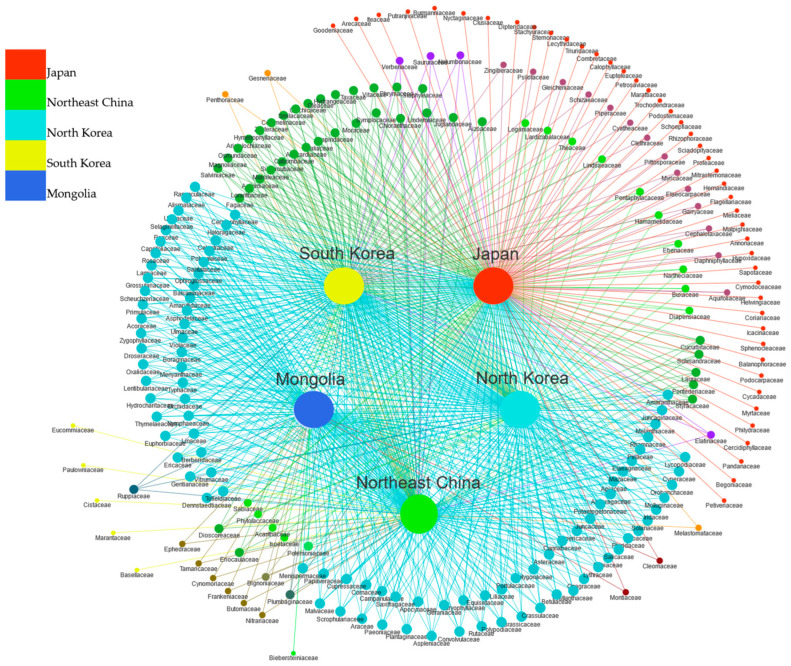
The results of a similarity and local endemism analysis for all families in five regions: Japan, Northeast China, North Korea, South Korea, and Mongolia. The dots connecting multiple rays indicate that a particular family is shared by several countries.

In this article, “locally endemic” refers to taxa that are unique to a single region within Northeast Asia, although they may also occur outside the area. For the families that compose the flora of Northeast Asia, 94 families are common to the five regions, accounting for 41.04% of the total families. Japan has the largest number of locally endemic families, at 45, accounting for 20.83% of the total number of families in Japan, including 113 species and infraspecific taxa, accounting for 1.77% of the total. Among them, 17 families are single-species families, and the remaining 28 families are small families. There is one locally endemic family in Northeast China, namely Cistaceae (1), accounting for 0.67% of the total families in China. There is one locally endemic family in Mongolia, Biebersteiniaceae (1), accounting for 0.93% of the total families in Mongolia. Neither North Korea nor South Korea has a locally endemic family (Figure 4).

#### 2.1.3. Analysis of the Components of the Genera

Based on the species (including infraspecific taxa) richness of genera, five genus levels have been classified: ultra-large genera containing more than or equal to 50 species; large genera containing 20 to 49 species; medium genera containing from 10 to 19 species; small genera containing from 2 to 9 species; and single-species genera containing only one species. The 1782 genera in Northeast Asia are classified according to the above levels (Table 2). 

In Northeastern Asia, there are 8 ultra-large genera that each have a minimum of 100 species and infraspecific taxa, which are *Carex* (420), *Artemisia* (158), *Astragalus* (146), *Saussurea* (128), *Salix* (117), *Oxytropis* (117), *Cirsium* (115), and *Viola* (114). Japan and Northeast China are characterized by the presence of *Carex* (275), *Cirsium* (100), and *Carex* (173) *Artemisia* (106), respectively. While North Korea, Mongolia, and South Korea are each host to only one such genus, which are *Carex* (153), *Astragalus* (118), and *Carex* (160), respectively.

The large genera contribute the second most to the flora of Northeast Asia, with 27 genera, accounting for 25.31% of total species, after the small genera, which contain 748 genera, accounting for 27.15% of total species. Single-species genera contribute the least with the highest number of genera, that is 792 genera, accounting for 7.55% of total species. While large genera and medium genera contribute differently, which are 83 genera, accounting for 23.43% of total species, and 132 genera, accounting for 16.55% of total species, respectively.

In contrast, in the five regions of Northeast Asia, small genera contributed the largest number of species to their respective flora. Furthermore, with different genus levels together, the small genera constitute the main composition of their floras. In North Korea and South Korea, it is medium genera that together with the small genera contribute 67.52% and 68.36% of the flora, respectively. In Japan and Northeast China, it is large genera that together with the small genera contribute 55.31% and 60.64%; while in Mongolia, it is the ultra-large genera that together with the small genera contribute 61.58% of the total.

**Figure 5:**
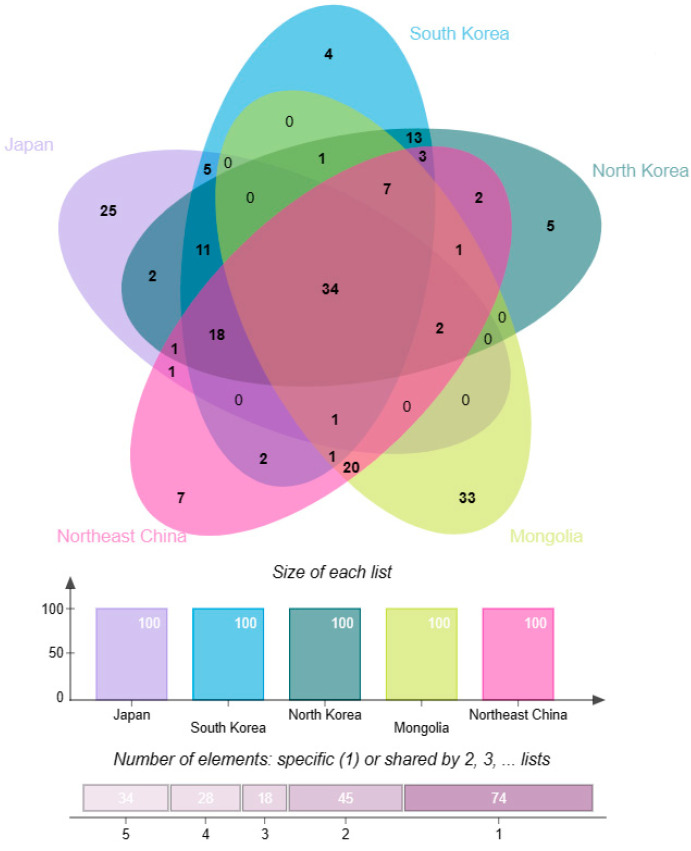
Similarity analysis of the dominant genera in five regions: Japan, Northeast China, North Korea, South Korea, and Mongolia. The size of each list represents the number of dominant genera, and the number of elements indicates the number of dominant genera that are either dominant to one region or shared by two, three, four, or five regions. The number at each intersection shows the number of shared dominant genera.

In this paper, we define the top 100 largest genera as the dominant genera in each region, given that the number of species contained in these genera is much higher than the average number of species contained in each genus in their respective regions. Among these dominant genera, thirty-four genera are common to all five regions, and *Carex* is the ultra-large genus with a high number of species across all five regions. Other commonly found genera include *Artemisia*, *Salix*, *Viola*, *Saussurea*, *Poa*, *Potentilla,* and *Galium*. Some genera, such as *Adenophora*, *Iris*, *Equisetum*, *Festuca*, *Lathyrus*, *Elymus*, and *Ligularia*, are small to medium in size in Japan but are dominant in four of the five regions in Northeast Asia. Similarly, *Euphorbia* is dominant in four regions except for North Korea (whereas small), as are *Primula* (small) and *Stellaria* (small) in South Korea (Figure 5).

The dominant genera in Mongolia differ greatly from those in the other four regions. In the remaining four regions, 18 genera are dominant, while in Mongolia they are either small genera, such as *Vincetoxicum*, *Rhododendron*, *Dryopteris*, *Rubus*, *Cyperus*, *Asplenium*, *Persicaria*, *Angelica*, *Prunus*, *Lonicera*, *Polygonatum*, *Lilium*, *Anemone*, *Betula*, and *Lespedeza*, single-species genera like *Euonymus* or have no distribution like *Quercus* and *Acer*.

*Dryopteris*, which have a high number of species in North Korea, South Korea, and Japan, are considered medium genera in China and small genera in Mongolia, respectively. *Astragalus* (118) and *Oxytropis* (97) are the largest genera in Mongolia and are also considered the large genus and middle genus in Northeast China, with 51 and 36 species and infraspecific taxa, respectively. However, they are considered medium genus and small genus in Japan, with eleven and six species and infraspecific taxa, respectively, and both are small genera in North Korea, with five and two species and infraspecific taxa, respectively. Finally, in South Korea, *Astragalus* is a small genus with four species and infraspecific taxa, while *Oxytropis* has no distribution.

**Figure 6:**
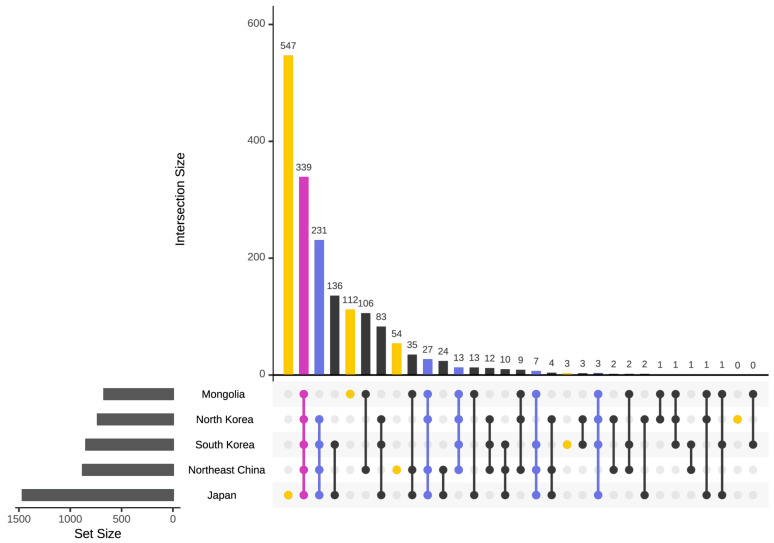
Similarity and local endemism analysis of all the genera in five regions, Japan, Northeast China, North Korea, South Korea, and Mongolia. Intersection size: the number of the shared genera; set size: the total number of the genera.

The floras of the five regions exhibit a high degree of similarity in terms of the genera that compose them, with 339 genera shared among all the regions. Except for the common genera in these five regions, among the four regions excluding Mongolia, Japan, Northeast China, North Korea, and South Korea exhibit the highest level of similarity, with as many as 231 genera shared. However, the remaining number of genera shared among the 4 regions is significantly lower, with 27, 13, 7, and 3, respectively (Figure 6).

The local endemism of genera across the five regions is characterized by significant variations. Japan has the highest number of locally endemic genera, with 547, accounting for 37.39% of the total genera in Japan. Mongolia follows, with 16.72% of its total genera being locally endemic which is 112 genera. Northeast China has 54 locally endemic genera, which constitute 6.16% of the total genera. In contrast, South Korea has only three locally endemic genera, namely *Abeliophyllum*, *Mankyua*, and *Triadica*, representing a mere 0.35% of the total genera in the country. North Korea, on the other hand, has no locally endemic genera.

Upon examining the distribution of shared genera in pairs of regions, South Korea and Japan exhibit a noteworthy number of shared genera, with 136 genera accounting for 16.08% and 9.30% of the total genera present in their respective regions. Meanwhile, Northeast China and Mongolia exhibit a substantial presence of shared genera, with 106 genera representing 12.10% and 15.82% of their total genera. There is a modest sharing of genera between Northeast China and Japan, with 24 genera accounting for 2.74% and 1.64% of their total genera. Additionally, Mongolia and Japan share 13 genera, while North Korea and South Korea share 3 genera, including *Megaleranthis*, *Hanabusaya*, and *Pentactina*. The shared genera are limited between North Korea and Northeast China, with only two genera being shared, *Carlesia* and *Physocarpus*. *Bergenia* are shared between Mongolia and North Korea; *Platycladus* are shared between South Korea and Northeast China; *Andromeda* and *Honckenya* are shared between North Korea and Japan. However, no common genera are shared between Mongolia and South Korea.

#### 2.1.4. Analysis of the Components of the Species and Infraspecific Taxa

**Figure 7:**
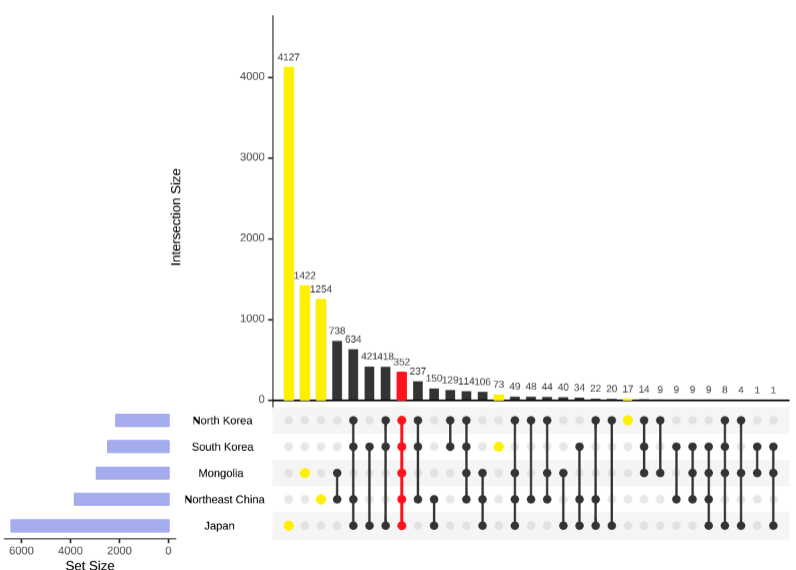
Similarity and local endemism analysis of all the species and infraspecific taxa in five regions, Japan, Northeast China, North Korea, South Korea, and Mongolia. Intersection size: the number of the shared species and infraspecific taxa; set size: the total number of the species and infraspecific taxa.

The flora of Northeast Asia comprises 10,514 species, including infraspecific taxa, distributed across five regions: Northeast China, North Korea, South Korea, Mongolia, and Japan. Among them, 352 species and infraspecific taxa are shared across all 5 regions, indicating a long history of biogeographical connection. Considerable variations in species richness and local endemism were observed among the different regions. Japan stands out as the most species-rich region with a total of 6395 species, of which 4127 are locally endemic. Northeast China takes second place with 3810 species, of which 1254 are locally endemic. Mongolia, with 2920 species, ranks third in terms of total species, and with 1422 locally endemic species, ranks third in terms of local endemism. The lowest and closest numbers of species were recorded in South Korea and North Korea with 2463 and 2119 species, respectively, and similarly, these 2 regions also exhibit the lowest levels of local endemism, with 73 and 17 species, respectively (Figure 7).

**Figure 8:**
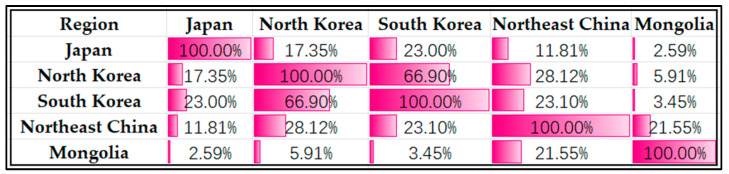
Similarity and statistics analysis of all the species and infraspecific taxa in five regions, Japan, Northeast China, North Korea, South Korea, and Mongolia.

The Jaccard (1901) [25] coefficient formula was employed to compute the similarity coefficients of species between the five regions of Northeast Asia: Japan, North Korea, South Korea, Northeast China, and Mongolia, as illustrated in Figure 8. The highest coefficient was observed between North Korea and South Korea, indicating that the composition of species between the two regions is more similar than that of the other regions, with a value of 66.90%. On the other hand, Japan and Mongolia exhibited the lowest similarity, with a coefficient of only 2.59%. The similarity coefficient for Northeast China was found to be relatively lower in comparison to North Korea and South Korea, yet still exhibited a moderate level of similarity with the other regions. Notably, the three lowest values of similarity coefficient are observed in the comparisons between Mongolia and the other three regions, except for Northeast China, which presents a higher similarity coefficient with Mongolia.

### 2.2. Statistics and Analysis of the Geographical Components of the Genera

#### 2.2.1. Statistics and Analysis of the Geographical Components of the Seed Plant Genera in Northeast Asia

Based on the areal classification system proposed by Wu [26] for seed plant genera in China, the 1686 genera of seed plants found in Northeast Asia exhibit 14 distribution patterns (Table 3 and Table 4). The north temperate distribution pattern dominates, with 327 genera representing 19.40% of all seed plant genera in the region, and a total of 3496 species and infraspecific taxa, accounting for 36.22% of all seed plants in Northeast Asia (9649). In contrast, the Pantropic distribution pattern is composed of 230 genera, but only 864 species and infraspecific taxa, accounting for 8.95% of the total number of seed plants. The cosmopolitan distribution pattern includes 114 genera and 2224 species and infraspecific taxa, making up 23.04% of the total number of seed plants. These three distribution patterns together cover 39.80% of the genera and 68.21% of the species and infraspecific taxa found in Northeast Asia. Other distribution patterns with more than 100 genera include the East Asia distribution pattern, the old-world temperate distribution pattern, East Asia and North America disjunct distribution pattern, and the Tropical Asia and Tropical Australasia distribution pattern, accounting for 11.98%, 9.96%, 6.17%, and 6.47% of the total genera, respectively. These 7 distribution patterns encompass the vast majority (74.38%) of all seed plant genera, 8536 species, representing 88.43% of all seed plants in Northeast Asia. The aforementioned seven distribution patterns also encompass the vast majority (70.37%) of all native plant genera, while accounting for 81.16% of the total species in Northeast Asia. Consequently, these seven distribution patterns can be considered the predominant patterns in terms of the geographical distribution of seed plants in this region, and they can provide a valuable basis for further understanding of the biogeographical and ecological processes that shape plant diversity in the region.

#### 2.2.2. Statistics and Analysis of the Geographical Components of the Genera in Five Regions

In these five regions, the North Temperate distribution pattern emerged as the most distinctive and widespread feature, exhibiting the highest number of genera. The genera observed in Japan and South Korea demonstrated pronounced tropical characteristics, as evidenced by the second highest number of genera belonging to the Pantropic distribution pattern in both countries. In contrast, the old-world temperate distribution pattern played a more significant role in the floristic composition of seed plant genera in Northeast China and North Korea than the pantropic distribution pattern. The characteristics of the pantropic distribution pattern were relatively weak in the westernmost region of Mongolia, while those of the old-world temperate distribution pattern were clearly evident. Furthermore, the East Asia distribution pattern displayed a decreasing trend in the number of genera with increasing longitude from east to west. Conversely, the cosmopolitan distribution pattern showed relatively consistent representation across the five regions in terms of the number of genera it contained.

### 2.3. Distribution of Species and Infraspecific Taxa of the Northeast Asia Flora

#### 2.3.1. Distribution of Species and Infraspecific Taxa of the Northeast Asia Flora

**Figure 9:**
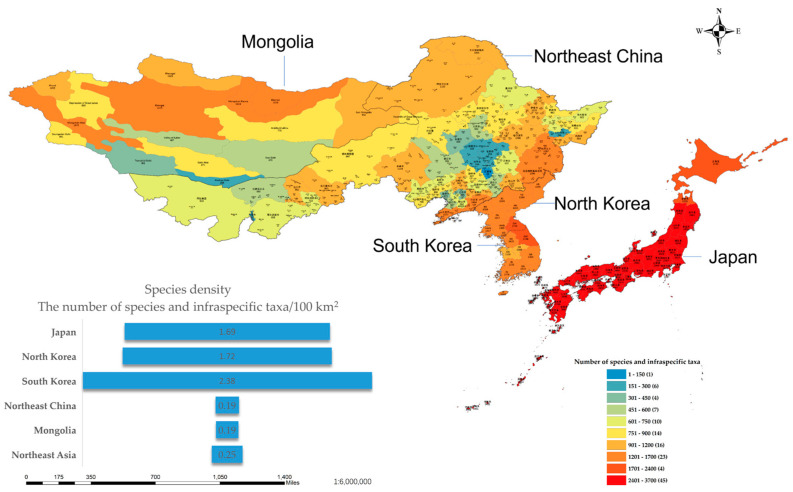
The distribution of species and infraspecific taxa in Northeast Asia (including Japan, Northeast China, North Korea, South Korea, and Mongolia) and their density, represented by the number of species and infraspecific taxa per hundred square kilometers. The distribution map classifies the number of species and infraspecific taxa into 10 categories, ranging from 0 to 2100. The range of 1–900 is evenly divided into 6 categories, while the other 4 categories are 901–1200, 1201–1700, 1701–2400, and 2401–3700, with the number of areas within each category indicated in parentheses. The color of each category progressively changes to red as the number of species increases. The length of the bar in the figure indicates the species density, where a longer bar corresponds to a higher number of species and infraspecific taxa per hundred square kilometers.

Through the integration of botanical literature and specimens, we have significantly enhanced the precision of the species distribution in Northeast Asia (Figure 9). Specifically, the distribution of species in Japan can be delineated to as accurate as 47 first-level administrative units. In China, we have mapped the distribution of species to a fine scale, encompassing 49 municipal administrative districts across four provinces. In North Korea and South Korea, the distribution is delineated, respectively, to ten and nine first-level administrative units. For Mongolia, the distribution of species is mapped to 16 phytogeographical regions. The distribution map depicts a cumulative count of the number of species observed within each administrative unit, which is indicative of the region’s richness of species diversity and correlates with the population size of each species. The data’s accuracy is, naturally, affected by the level of research performed in each area. Moreover, we have calculated the species density per hundred square kilometers for each region based on the total area and number of species in the region (Figure 9). This measure reflects the mean species richness and serves as a direct indicator of the level of biodiversity in each region.

The Northeast Asian region encompasses a vast area with an overall species density that is higher than that of Mongolia and Northeast China but much lower than that of Japan, North Korea, and South Korea. From the distribution map, it can be observed that species distribution in Northeast Asia is uneven and generally exhibits a decreasing trend from east to west. Due to its status as an island country, Japan boasts not only a high species density but also a very high population number, making it a veritable hotspot of species distribution on the map. Located on the Korean Peninsula, North Korea and South Korea have lower species numbers than Northeast China and Mongolia but a much higher species density, placing them second only to Japan in terms of distribution hotspots on the map. Northeast China, on the other hand, has a relatively low species density and a dispersed population distribution, with only the Liaodong Peninsula, Changbai Mountain, and Xiaoxing’anling showing relatively concentrated populations. As the second largest landlocked country in the world, Mongolia has a low species density, with only hotspots in areas with developed water systems, such as the Selenge River Basin in the north and Har Us Lake in the west.

#### 2.3.2. Distribution of Locally Endemic Species and Infraspecific Taxa across the Five Regions

**Figure 10:**
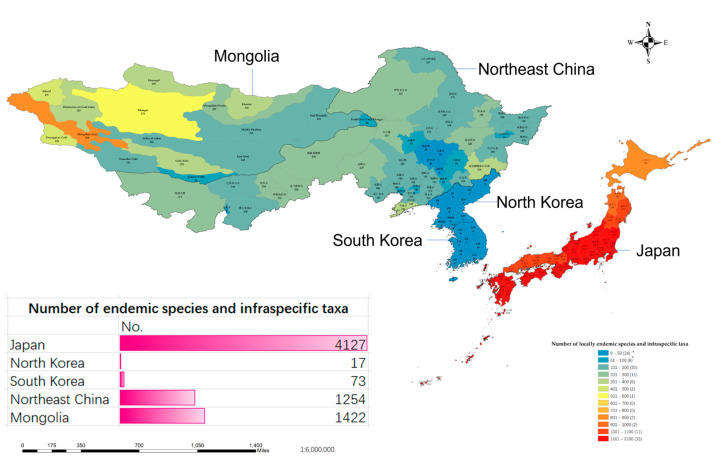
The distribution of locally endemic species and infraspecific taxa in Northeast Asia (Japan, Northeast China, North Korea, South Korea, and Mongolia) and their total count. The distribution map classifies the number of species into 13 categories, ranging from 0 to 2100. The first two categories include species counts from 0–100, while the range of 101–1100 is evenly divided into 10 categories. The final category covers species counts from 1101–2100. The color of each category becomes progressively closer to red as the number of species increases. The length of the bar in the figure represents the total count, whereas a longer bar indicates a higher count of locally endemic species and infraspecific taxa.

Focusing on the distribution of locally endemic species within the region (Figure 10), Japan emerges as the foremost hotspot of species distribution in Northeast Asia, with a significantly higher number of locally endemic species and aggregation degree than the other four regions. North Korea and South Korea display an exceedingly high similarity in species composition, resulting in the lowest number of locally endemic species among the five regions. Although Northeast China does not have a low number of locally endemic species, they are more dispersedly distributed without any particularly aggregated hotspots. Mongolia exhibits a roughly equivalent distribution of locally endemic species to Northeast China, with its most obvious aggregation hotspots occurring in close proximity to the Altai Mountains, the highest range in the northwest.

### 2.4. The Relationship between Species Distribution Patterns and Climate in Northeast Asia

#### 2.4.1. The Dominant Factor in Classifying the Climate Types of Northeast Asia: Continentality

**Figure 11:**
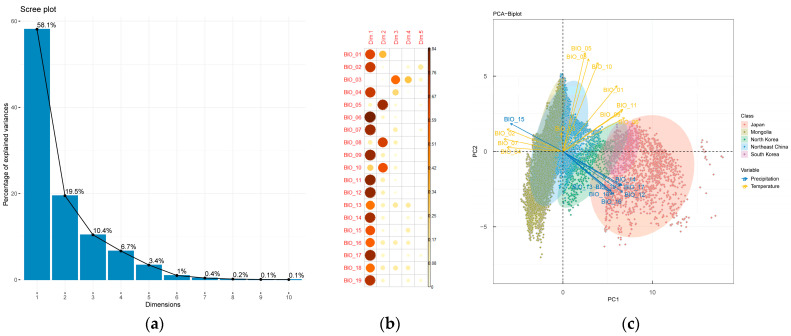
(**a**) This scree plot describes the contribution rate of each principal component, where the curve rapidly drops at the second principal component. Therefore, the first and second principal components were selected. (**b**) The quality of factors, represented by the square of loadings, indicates the degree to which a principal component represents a given variable. Higher factor quality values indicate that the principal component represents the variable more accurately. (**c**) Spatial gridding was conducted on five regions in Northeast Asia, resulting in solid rhombuses that represent the distribution of all grids in the principal component space. The red arrows indicate 8 precipitation factors (BIO12–BIO19) while the blue arrows indicate 11 temperature factors (BIO1–BIO11). The proximity of the rhombuses to the arrows reflects the magnitude of the values. The confidence interval of the confidence ellipse is 95%.

By utilizing the dataset comprising 19 bioclimatic variables obtained from WorldClim [27] during the period spanning from 1970 to 2000 (Appendix A), the Northeast Asia region was spatially divided into a grid with a spatial resolution of 10 min. Subsequently, 16,844 bioclimatic factor data points were extracted and subjected to principal component analysis. Principal components 1 and 2, with contribution rates of 58.13% and 19.51%, respectively (see Appendix B and Appendix C), were selected for further analysis (Figure 11). Fifteen bioclimatic variables related to temperature and precipitation have high loading values on the first principal component, including annual mean temperature, mean diurnal range, temperature seasonality, minimum temperature of the coldest month, temperature annual range, mean temperature of the driest/coldest quarter, annual precipitation, precipitation of the wettest/driest month, precipitation seasonality, and precipitation of the driest/wettest/warmest/coldest quarter. Three bioclimatic variables have high loading values on the second principal component, which are the maximum temperature of the warmest month and the mean temperature of the wettest/warmest quarter. Together, the first and second principal components adequately represented almost all bioclimatic variables, demonstrating their importance in understanding the bioclimatic conditions of the Northeast Asia region (Figure 11).

Based on the principal components analysis, five regions in Northeast Asia show significant discrete relationships along the two directions of principal components 1 and 2 (Figure 11). Japan and South Korea exhibit the closest climate patterns, as reflected in their high min temperature in the coldest month and mean temperature in the driest and coldest quarters. Moreover, these regions have the highest precipitation under all indicators, with the smallest seasonality. On the other hand, North Korea, Northeast China, and Mongolia exhibit independent climate patterns, with overlapping features that reflect a transitional relationship. North Korea is closer to Japan and South Korea in terms of precipitation and lowest temperature, whereas Northeast China and Mongolia are closer to each other in terms of the highest temperature indicators, both of which are far higher than those of Japan, South Korea, and North Korea. Notably, Mongolia has a significantly higher mean diurnal range, temperature annual range, and temperature seasonality than the other four regions.

**Figure 12:**
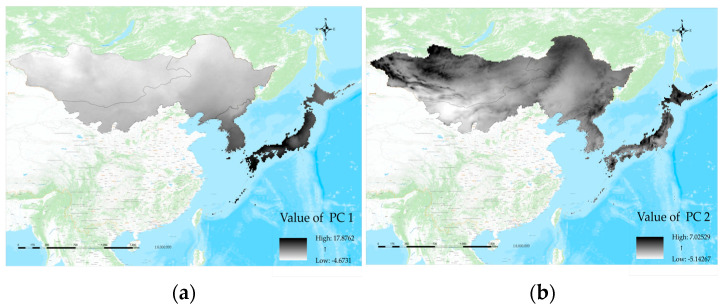
(**a**) The principal component 1 (PC1) reflects significant “continental” characteristics. As the longitude decreases, the value of PC1 decreases, the color becomes brighter, and the continental features become stronger. (**b**) The principal component 2 (PC2) reflects significant “altitude” characteristics. At the same longitude, the higher the value of PC2, the deeper the color and the higher the altitude.

When projecting the values of principal components 1 and 2 back onto the map of Northeast Asia, a climate distribution pattern emerges that is consistent with geographical location and conforms to the spatial pattern of continentality (Figure 12a). The intricate relationship between climate and geography underscores the fact that geographic factors can affect climate patterns, while climate patterns can also influence geographical phenomena, vegetation, and ecosystems [28,29]. One of the expressions of that intricate relationship is the feature of continentality. The pronounced “Continentality” feature reflected by principal component 1 increases from east to west, with lower PC1 values and geographical locations further away from the ocean and deeper into the center of the continent. The climatic characteristics in these areas show a gradual decrease in precipitation and humidity, an increase in temperature variation, and a large diurnal temperature difference, with smaller nocturnal temperature differences. Additionally, maximum and minimum temperatures become more polarized. Japan, as an island country, exhibits the least obvious continental features, while South Korea, located at the southern end of the Korean Peninsula, displays significant oceanity features as well. Moving from North Korea through Northeast China to Mongolia, continentality features become increasingly typical. On the other hand, PC2 reflects altitude and topographic characteristics, highlighting the mountainous distribution in Northeast Asia (Figure 12b). These areas include the high coastal mountains in Northwestern Japan, the hills in Northern Korea, the Greater Khingan Range (Da Hinggan Ling), Lesser Khingan Range (Xiao Hinggan Ling), and Changbai Mountains in Northeastern China, as well as the Altai Mountains region on the westernmost side of the Mongolian Plateau. In regions with high PC2 values where the altitude is higher, the climate tends to be drier, colder, and more extreme.

#### 2.4.2. The Relationship between Species Distribution Patterns and Continental and Altitude Factors in Northeast Asia

**Figure 13:**
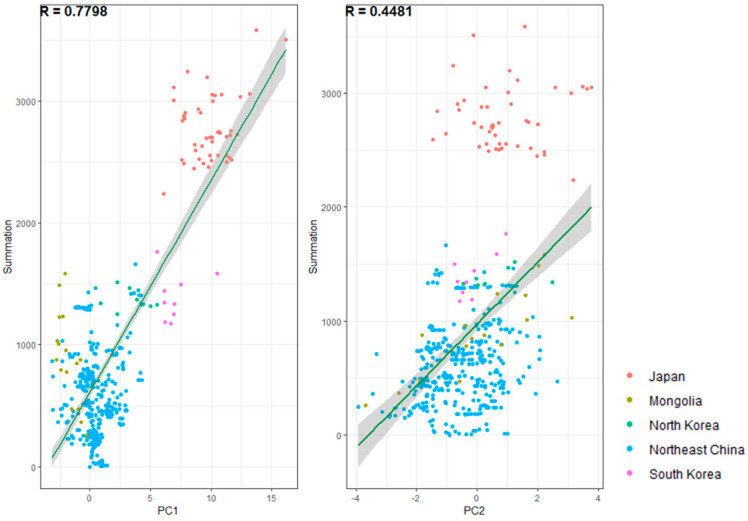
Pearson correlation analysis was performed between 472 pieces of species number data and $\bar{PC1}$, $\bar{PC2}$ values, yielding correlation coefficients of R_1_ = 0.7798 and R_2_ = 0.4481 (95% confidence interval). Y-axis (summation): the number of species and infraspecific taxa associated with each distribution unit; X-axis (PC1 and PC2): the average values of PC1 and PC2 associated with each distribution unit.

To elucidate the dominant factors that affect the distribution pattern of species, a Pearson correlation analysis was conducted utilizing the values of main components 1 and 2 in Northeast Asia in conjunction with species distribution data. The dataset comprises species distribution information within Japan’s 47 first-level administrative regions, 19 first-level administrative divisions on the Korean Peninsula, 390 county-level administrative regions in Northeast China, and 16 phytogeographical regions in Mongolia, totaling 472 pieces of species number data (summation). For each division, average values of PC1 and PC2 were obtained at a spatial resolution of 10 min, yielding $\bar{PC1}$ and $\bar{PC2}$. The dataset was subjected to normal distribution tests, and Pearson correlation analysis was performed utilizing Summation and $\bar{PC1}$, as well as summation and $\bar{PC2}$ (Figure 13). The results indicate a statistically significant correlation between the number of species and the value of $\bar{PC1}$, which suggests a close relationship between species distribution patterns and continental characteristics. Conversely, a mere general correlation exists between the number of species and the value of $\bar{PC2}$, which indicates a feeble connection between the formation of species distribution patterns and altitude and topographic factors characteristics. Significantly, the summation data demonstrate that Japan has a greater number of species and deviates from the summation-$\bar{PC2}$ regression line. This highlights that in island areas where continental characteristics are least significant, the relationship between species distribution patterns and altitude characteristics is weakest.

### 2.5. Threatened Species Situation in Northeast Asia

#### The Threatened Species List of Five Regions in Northeast Asia

**Figure 14:**
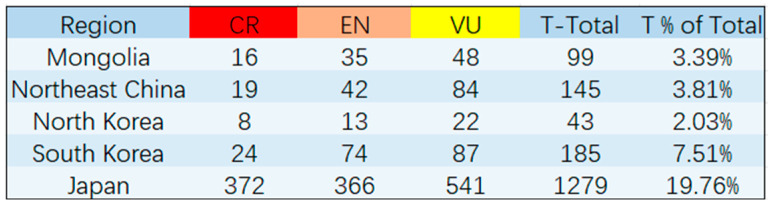
The number of threatened species in five Northeast Asia regions. IUCN Red List of Threatened Species is a grouping of three categories: critically endangered (CR), endangered (EN), and vulnerable (VU). T-Total: threatened species total; T % of Total: the percentage of threatened species out of the total number of species.

In this study, we conducted a statistical analysis of the situation of IUCN-threatened species in five regions of Northeast Asia, namely North Korea, South Korea, Japan, Northeast China, and Mongolia. To achieve this, we referred to the national red list research literature published by these regions [30,31,32,33,34]. The results of this analysis are presented in Figure 14. Our findings reveal that Japan has a significantly higher number of threatened species compared to the other four regions. Conversely, South Korea, North Korea, and Mongolia have similar total numbers of species but exhibit significant differences in the number of threatened species. China conducted a comprehensive assessment of all 35,784 species present within the country and identified 3879 species as threatened nationwide [32]. Notably, a meager 145 of these threatened species are exclusive to Northeast China.

**Figure 15:**
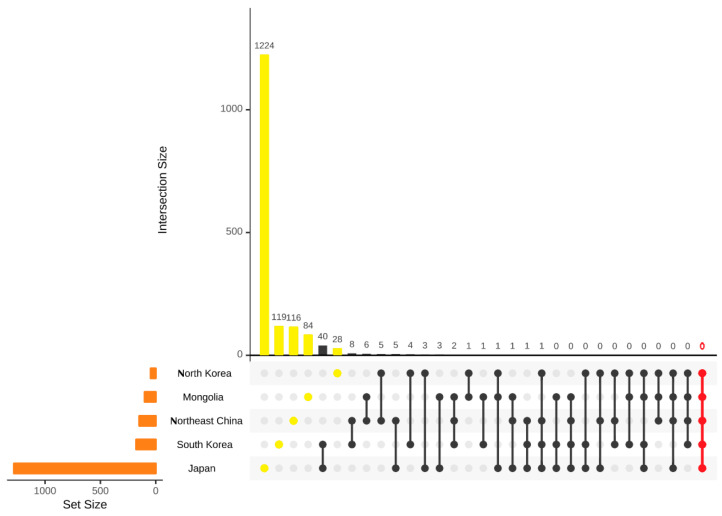
Similarity analysis of the threatened species in five regions, Japan, Northeast China, North Korea, South Korea, and Mongolia. Intersection size: the number of the shared threatened species and infraspecific taxa; set size: the total number of the threatened species and infraspecific taxa.

The current study investigates the extent of sharing of threatened species across five Northeast Asia regions: North Korea, South Korea, Japan, China, and Mongolia (Figure 15). While no threatened species are shared among all five regions, three threatened species—*Cypripedium guttatum* is considered as a threatened species by North Korea, South Korea, Japan, and China but not by Mongolia. Nevertheless, there are variations in the classification of these species across the regions. For instance, *Cypripedium guttatum* is classified as critically endangered (CR) in Japan and South Korea, vulnerable (VU) in North Korea and China, and not considered a threatened species in Mongolia. Similarly, *Epipogium aphyllum* is categorized as vulnerable (VU) in Mongolia, and endangered (EN) in Japan and China, while not considered threatened in North Korea and South Korea. *Cypripedium macranthos* is categorized as endangered (EN) in China and South Korea, critically endangered (CR) in Japan, and not considered threatened in North Korea.

Furthermore, only a few threatened species are shared among the three countries. *Pinus pumila* is considered EN species in Mongolia, VU species in South Korea, and CR species in China, but not in Japan and North Korea. *Rhododendron aureum* is considered VU species in Mongolia and China, while EN species in South Korea. Lastly, *Drosera anglica* is considered EN species in Mongolia and North Korea, and VU species in Japan, but not in South Korea, and is absent from Northeast China.

Our analysis revealed that Japan and South Korea have 40 species of threatened species in common, while the other three countries have a limited number of shared threatened species in pairs of regions. In terms of threatened species locally endemic to each country, Japan has the highest number with 1224 species, followed by South Korea and Northeast China with 119 and 116 species, respectively. Mongolia has 84 locally endemic threatened species, and North Korea has the lowest number, with only 28 species.

The number of threatened species in the five regions exhibits significant variation, which may be attributed to differences in species richness or three other factors. First, the level of development may play a role, as developed countries with higher levels of industrialization, such as Japan and South Korea, tend to have more threatened species. North Korea, despite having a similar species composition, has only a quarter of the threatened species compared to South Korea. Second, the variation may be due to differences in the depth of national assessments, which directly impact the number of threatened species. Third, the variation may reflect differences in the resilience of local ecosystems, as a higher proportion of threatened species implies poorer ecosystem resilience and the need for higher resistance to interference and sustainability standards.

## 3. Discussion

### 3.1. The Tropical Composition of Northeast Asia

The Northeast Asia region covers a total area of 4.1411 million km^2^ and spans from 30° N to 49° N latitude. Despite its location in the northern temperate zone, the flora of Northeast Asia exhibits a relatively high proportion of tropical genera, comprising 35.29% of the total number of genera. Japan possesses the highest proportion of tropical genera at 42.25%, followed by South Korea at 29.25%, North Korea at 21.59%, Northeast China at 17.38%, and Mongolia with the lowest proportion at a mere 6.45%.

The extensive fossil records found in Northeast China illustrate a close relationship between the region’s flora and that of tropical areas, indicating that many tropical plants thrived in this area in the past. Studies based on fossil evidence suggest that Northeast China may have served as, or been close to, the center of origin and diversification of flowering plants [35,36]. Despite climate changes that forced tropical plants to migrate southward, the ancient and complex topography of Northeast Asia provided conditions for the retention of these tropical plants, resulting in a higher proportion of tropical elements in the Northeast Asian flora [37].

Furthermore, the tropical composition of the flora in Northeast Asia may also be attributed to its connection with the South Asian tropical flora. It is generally accepted that the South Asian tropics have extensive and close ties to the north temperate flora via East Asia, particularly Southern China [38]. This may be because East Asia was less affected by Quaternary glaciers and had a lower extinction rate for tropical plants during this period [37]. At present, East Asia’s climate and topography have made it a suitable environment for these plants to survive. As tropical plant distribution radiated from East Asia to its surroundings, such as the Korean Peninsula and Japan, these regions became good habitats for these tropical plants due to their similar precipitation and seasonal temperature patterns [39].

However, factors such as temperature, precipitation, and topography play critical roles in determining whether tropical plants can thrive. Compared to South Korea, North Korea has a less favorable environment for growing tropical plants due to its higher altitude and lower temperature, while Mongolia’s high altitude and cold, dry climate make it challenging for tropical plants to survive as well [12]. Additionally, mountain barriers between Mongolia and Northeast China may also impede the spread of tropical plants, explaining why Mongolia’s flora has a low proportion of tropics.

Finally, it is worth considering the potential role of shallow seas in influencing plant diversity, which may involve factors such as the creation of isolation zones, the impact of sea level fluctuations on species production, and the effects of warm ocean currents on regional climate and vegetation distribution [40,41]. This may help explain why certain species-rich super-genera have formed in the floras of Japan and South Korea [42], including but not limited to genera of tropical elements such as the cosmopolitan genus *Carex*, tropical genera *Prunus* and *Fimbristylis*. The shallow sea areas that lie between mainland China, the Korean Peninsula, and Japan may have facilitated the development of this effect.

The high proportion of tropical plants in the flora of Northeast Asia can be attributed to several factors. Firstly, residual tropics have been preserved in Northeast Asia due to its geological history. Secondly, there is a possibility of radiation from Southeast China’s tropics. Finally, small-scale habitat specificity caused by topography and sea level fluctuations also contributed to this feature. Ultimately, Eastern China played a vital role in linking north–south and east–west connections, contributing to the distinct feature of Northeast Asian flora with a high proportion of tropical genera.

### 3.2. The Large-Scale Spatial Patterns of Plant Diversity in Northeast Asia

Our current understanding of large-scale spatial patterns of plant diversity is primarily based on floristic books or visualizations of stacked distribution range maps. While these methods accurately quantify the total number of species present in a region, they fall short of elucidating the mechanisms of species coexistence or the formation of species-rich or species-poor communities [43,44,45,46]. Furthermore, spatial grain, which refers to the unit area used to count species numbers, exhibits a nonlinear correlation with alpha diversity, i.e., the species richness of local plant communities [47,48,49,50]. This nonlinear relationship impedes the direct comparison of biodiversity data from different grains, posing a challenge in inferring large-scale patterns of α diversity [51]. Consequently, the study of broad-scale spatial patterns of biodiversity necessitates a trade-off between spatial grain and extent, in spite of emphasizing the need to unify spatial grains [50]. Even in well-sampled regions, the final species data are a heterogeneous mixture of surveys that integrate different spatial grains, protocols, and taxonomic criteria [51,52,53].

In this article, the distribution range used to count the number of species can be seen as different spatial grains of varying sizes. For example, in Japan, there are 47 first-level administrative regions, while North Korea and South Korea have 10 and 9 regions, respectively. Northeast China has 390 county-level administrative regions, and Mongolia has 16 phytogeographical regions, resulting in a total of 472 grains. While areas, where species-rich and species-poor communities occur, may vary under different spatial grains, in plant diversity hotspots such as East Asia, the range of hotspots marked by different grains under forest environments is not significantly different [51]. However, under non-forest environments, such as grasslands, the larger the grain size, the more accurate the hotspot range [51]. Therefore, although there is a considerable difference in the range used for species counting among the five regions in Northeast Asia, with Mongolia having the largest range of species counting units, the species data are still comparable since Mongolia has more grasslands that are suitable for larger size grains.

**Figure 16:**
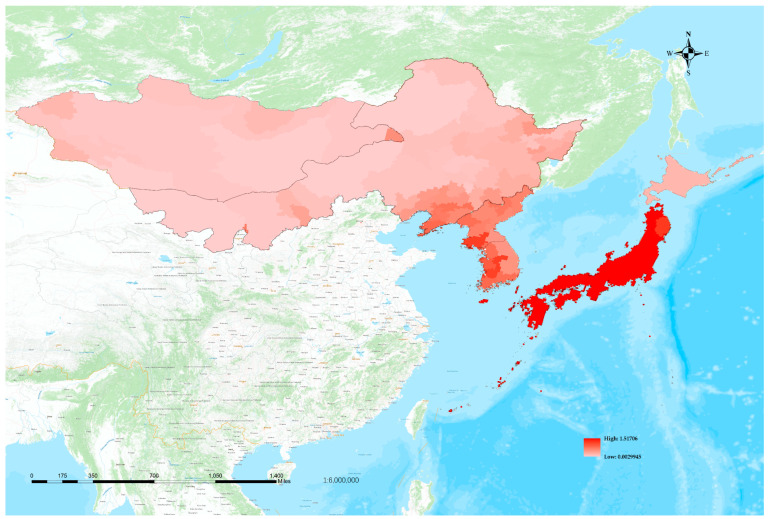
Species diversity hotspots map created using a measure calculated by dividing the number of species in each area by its corresponding range area. The intensity of red coloration reflects higher species density per square kilometer.

To further reduce the impact of range heterogeneity, we use species density per square kilometer, which is calculated by dividing the number of species and infraspecific taxa by the corresponding range area. We use this measure to draw species diversity hotspot maps, which will facilitate our discussion on the causes of plant diversity patterns (Figure 16).

The figure (Figure 16) clearly depicts that the plant diversity hotspots in Northeast Asia can be categorized into three gradients. The first gradient is Japan, with the exception of certain regions in Hokkaido. The second gradient encompasses the Korean Peninsula and the coastal areas of Northeast China. The third gradient comprises Hokkaido, the inland regions of Northeast China, and Mongolia. This evident gradient relationship is a distinctive characteristic feature of species distribution patterns in Northeast Asia.

It is widely acknowledged that the distribution pattern of plant species is influenced by plant adaptation and their interaction with both biotic and abiotic environments [54,55]. At larger spatial scales, ranging from 10 km^2^ to global extents, abiotic environmental factors are believed to be the primary determinants of plant species distribution patterns [54,56]. The latitudinal diversity gradient pattern (LDG), which describes a gradual decrease in biodiversity with increasing latitude, is one of the most prevalent and readily observable biodiversity geographic distribution patterns on Earth [57]. Apart from latitude, other abiotic environmental factors, such as historical factors, climate, climate variability, geology, and topography, that impact regional-scale biodiversity distribution patterns, also need to be thoroughly considered [51,58].

The flora of Northeast Asia is widely recognized to have been shaped by two fundamental gradients, namely the latitude gradient and the continentality gradient [59,60]. Along the latitude gradient, the dominant environmental factors that shape diversity distribution patterns vary. In the northern temperate region, solar radiation is the main determinant, and temperature variables such as annual mean temperature and mean diurnal range play a dominant role [55]. These factors are reflected in the plant flora of Northeast Asia, which is divided into two major areas by the 40° N parallel. The first and second gradients are primarily distributed south of 40° N and represent species hotspots, while the third gradient, which represents species-poor areas, is mostly distributed north of 40° N.

Moving inland from the Pacific coast, the influence of the marine climate gradually diminishes, and the characteristics of the continental climate become more prominent. The first principal component (PC1) representing continental characteristics reveals three distinct gradients on the map. The first gradient is Japan, which is directly influenced by the Pacific Ocean, and has the weakest continental characteristics. The second gradient is the Korean Peninsula and the Liaodong Peninsula, which are mainly influenced by the Japan Sea, the Yellow Sea between the Liaodong Peninsula and the Korean Peninsula, and the Bohai Sea to the west of the Liaodong Peninsula, and where continental characteristics begin to increase. The third gradient encompasses Hokkaido, areas north of 40° N in Japan, as well as the inland regions of Northeast China and Mongolia, which are farther away from the ocean. In these locations, continental characteristics are more pronounced. 

The impact of continental characteristics on diversity distribution patterns is more pronounced in relatively low-latitude areas [58]. Consequently, the diversity distribution pattern of Northeast Asia approximately conforms to the continental gradient. However, several factors, such as altitude, topographic and minimum temperature, as represented by the second principal component (PC2), also have a significant impact on species distribution within the gradient. For instance, South Korea has more plain regions, whereas North Korea is dominated by mountains. The marine climate has a greater impact on the plain areas, making South Korea more similar to Japan than North Korea in terms of continental characteristics. The species diversity in South Korea, featuring a milder and humid climate is also relatively higher than that observed in North Korea. In Hokkaido, a region situated in high-latitude areas with lower minimum temperatures, species diversity is low. Furthermore, numerous mountains in Northeast China impede the penetration of marine influence and contribute to reduced species richness due to the increased elevation.

The distinctive continental features of Northeast Asia, such as its temperate climate and high precipitation, may have exerted a significant influence on the historical distribution patterns of its plant species. Climate models of the last glacial maximum are utilized to glean insights into the vegetation historical conditions of this region. The reconstructions have revealed that during this period, temperate forests were distributed continuously along the Japan, Korean Peninsula, and China’s northeast coastal area, although this distribution has been interrupted in modern times [39,61]. As such, it is highly probable that the diversity patterns of Northeast Asia had already formed two gradients of inland and coastal during the last glacial maximum.

In general, the distribution pattern characteristics of flora in Northeast Asia are notably distinct. On one hand, the continental features play a dominant role in differentiating the climate forms of the five regions, thereby giving rise to diverse patterns of species diversity distribution. On the other hand, the developmental history of this flora indicates that Northeast Asia’s five regions represent indivisible integrity.

## 4. Materials and Methods

To compile the species lists and corresponding distribution information, we gathered data from national and local flora and literature materials published in multiple countries. Simultaneously, we referred to these sources to exclude cultivated and alien species from the records. They are the “Flora of Japan” (1993–2020) [62,63,64,65,66,67,68], “Provisional checklist of vascular plants for the Korea Peninsula Flora (KPF)” [69], “The Genera of Vascular Plants of Korea” [70], “Conspectus of Flora of Outer Mongolia” (2014 edition) [71], “China Species 2000” [72], “Flora Plantarum herbacearum chinae borealieorientalis” [73], “Atlas of Northeast Plant Distribution” [74], “Inventory of species diversity of Liaoning higher plants” [75] and “Flora Intra Mongolica” (the third edition) [76]. For a small number of plant names, we employed the Taxonomic Name Resolution Service (TNRS) [77] to correct the spelling, while the majority of accepted names and authors were primarily standardized using POWO [78] online database. Additionally, we referred to WFO [79], TPL [80], Tropicos [81], and expert opinions for further guidance. We used the APG IV system to recognize the families [82].

All Wayne diagrams involved in this study were completed using the EVenn online mapping tool [83]. All species distribution maps involved in this study were produced using ArcGIS 10.2 software. We Downloaded 19 BIO climate factor variables (bio 10 m) from 1970 to 2000 on the Worldclim website [27], used ArcGIS 10.2 software to spatially rasterize the climate factor data within the research scope, converted the raster data set into point elements, and extracted the corresponding coordinates, climate factor data, and species distribution data for subsequent analysis.

In principal component analysis and correlation analysis, we used the stats package of R4.2.2 software, and in our drawing used the ggplot2 package [84].

The list of threatened species is from the Red List published by various countries [30,31,32,33,34].

$s=\frac{c}{a+b+c}$, The similarity coefficient calculation formula uses this equation, where *S* is the similarity coefficient between the two faunas, *c* is the common species of the two floras, and *a* and *b* are locally endemic species of the two regions, respectively [25].

This study has generated a catalog of the flora of Northeast Asia, which can be found in Supplementary Materials Dataset S1.

## Figures and Tables

**Table 1:** Distribution of ferns, lycophytes, gymnosperms, and angiosperms at family, genus, and species (including infraspecific taxa) levels in Northeast Asia and the other five regions.

Taxon Level	Plants	Japan	North Korea	South Korea	Northeast China	Mongolia	Northeast Asia
Family	Ferns	17	11	15	10	6	17
	Lycophytes	3	3	3	2	2	3
	Gymnosperms	6	2	3	3	3	7
	Angiosperms	190	131	144	134	96	198
	Sum.	216	147	165	149	107	225
Genus	Ferns	90	37	57	33	17	91
	Lycophytes	5	4	5	3	2	5
	Gymnosperms	16	6	9	9	6	19
	Angiosperms	1352	684	774	831	645	1667
	Sum.	1463	731	845	876	670	1782
Species and infraspecific taxa	Ferns	764	114	225	113	40	810
	Lycophytes	43	17	19	20	6	55
	Gymnosperms	44	14	19	45	21	91
	Angiosperms	5544	1974	2200	3632	2853	9558
	Sum.	6395	2119	2463	3810	2920	10,514

**Table 2:** Distribution of 5 genus levels in the flora of Northeast Asia and the other 5 regions. Ultra-large genera: ≥50 species and infraspecific taxa; large genera: 20–49 species and infraspecific taxa; medium genera: 10–19 species and infraspecific taxa; small genera: 2–9 species and infraspecific taxa; single-species genera: 1 species or 1 infraspecific taxa.

Genus-Level	No.	Japan	North Korea	South Korea	Northeast China	Mongolia	Northeast Asia
Ultra-large genera	No. Gen.	11	1	1	4	7	27
	No. sp.	1024	153	160	382	570	2664
Large genera	No. Gen.	49	6	7	25	14	83
	No. sp.	1341	152	172	729	374	2466
Medium genera	No. Gen.	85	29	38	55	36	132
	No. sp.	1120	348	472	718	454	1730
Small genera	No. Gen.	604	312	352	395	319	748
	No. sp.	2196	1084	1213	1584	1228	2862
Single-species genera	No. Gen.	714	384	448	401	294	792
	No. sp.	714	384	448	401	294	792

**Table 3:** The percentage contribution of each areal type to the total number of species and infraspecific taxa in Northeast Asia.

	The Percentages of the Total Number of Species and Infraspecific Taxa					
Areal Types	South Korea	North Korea	Mongolia	Northeast China	Japan	Northeast Asia
1. Cosmopolitan	24.64%	27.37%	26.68%	26.51%	19.50%	21.14%
2. Pantropic	9.38%	7.50%	1.37%	4.65%	12.07%	8.21%
3. Trop. Asia and Trop. Amer. Disjuncted	0.97%	0.61%	0.14%	0.42%	1.41%	0.95%
4. Old World Tropics	2.07%	1.89%	0.89%	1.39%	3.25%	2.29%
5. Tropical Asia and Trop. Australasia	1.83%	1.04%	0.07%	0.45%	3.05%	1.95%
6. Trop. Asia to Trop. Africa	0.65%	0.61%	0.03%	0.50%	1.16%	0.79%
7. Trop. Asia (Indo-Malesia)	2.92%	1.79%	2.33%	1.76%	3.38%	2.87%
8. North Temperate	28.46%	33.41%	42.77%	37.85%	26.96%	33.24%
9. E. Asia and N. Amer. Disjuncted	4.91%	4.91%	1.68%	3.75%	3.94%	3.28%
10. Old World Temperate	8.00%	8.97%	12.81%	12.26%	5.80%	9.12%
11. Temp. Asia	0.93%	1.32%	2.64%	1.97%	0.47%	1.25%
12. Mediterranea, W. Asia to C. Asia	0.28%	0.33%	4.42%	1.71%	0.55%	1.70%
13. C. Asia	0.08%	0.14%	2.29%	1.02%	0.02%	0.77%
14. E. Asia	4.95%	3.92%	0.31%	2.28%	5.83%	4.21%
Total	90.09%	93.82%	98.42%	96.51%	87.38%	91.78%

**Table 4:** Areal types of the seed plant genera of the Northeast Asia.

	No. of Genera					
Areal Types	South Korea	North Korea	Mongolia	Northeast China	Japan	Northeast Asia
1. Cosmopolitan	88	86	85	95	104	114
2. Pantropic	110	81	26	82	223	230
3. Trop. Asia and Trop. Amer. Disjuncted	14	7	1	7	37	39
4. Old World Tropics	33	26	7	22	97	97
5. Tropical Asia and Trop. Australasia	32	16	2	11	106	109
6. Trop. Asia to Trop. Africa	8	6	1	10	34	37
7. Trop. Asia (Indo-Malesia)	32	13	5	14	81	83
8. North Temperate	211	226	242	265	271	327
9. E. Asia and N. Amer. Disjuncted	56	50	20	57	91	104
10. Old World Temperate	85	83	114	120	112	168
11. Temp. Asia	19	24	37	39	18	52
12. Mediterranea, W. Asia to C. Asia	4	5	59	33	23	79
13. C. Asia	1	1	45	20	1	45
14. E. Asia	90	66	7	65	170	202
Total	783	690	651	840	1368	1686

## Data Availability

The data presented in this study are available in Dataset S1.

## Supplementary Materials

The following supporting information can be downloaded at: https://www.mdpi.com/article/10.3390/plants12122240/s1, Dataset S1: Catalog of the flora of Northeast Asia. It is a plain text file formatted in UTF-8 encoding, consisting of five columns: Column ID: a unique identifier for the TaxonName; Column TaxonName_with author: the scientific name of the taxon, including the authorship; Column Genus: the full scientific name of the genus in which the taxon is classified; Column Family: the full scientific name of the family in which the taxon is classified; Column Northeast Asia distribution: the co-occurrence of the plant across five regions of Northeast Asia (Japan, North Korea, South Korea, Northeast China, Mongolia), with regions separated by “|”.

## Appendix A

Meaning of the 19 bioclimatic factors (BIO).

BIO1 = Annual Mean Temperature (°C)

BIO2 = Mean Diurnal Range (Mean of monthly (max temp–min temp)) (°C)

BIO3 = Isothermality (BIO2/BIO7) (×100)

BIO4 = Temperature Seasonality (standard deviation × 100)

BIO5 = Max Temperature of Warmest Month (°C)

BIO6 = Min Temperature of Coldest Month (°C)

BIO7 = Temperature Annual Range (BIO5-BIO6) (°C)

BIO8 = Mean Temperature of Wettest Quarter (°C)

BIO9 = Mean Temperature of Driest Quarter (°C)

BIO10 = Mean Temperature of Warmest Quarter (°C)

BIO11 = Mean Temperature of Coldest Quarter (°C)

BIO12 = Annual Precipitation (mm)

BIO13 = Precipitation of Wettest Month (mm)

BIO14 = Precipitation of Driest Month (mm)

BIO15 = Precipitation Seasonality (Coefficient of Variation) (mm)

BIO16 = Precipitation of Wettest Quarter (mm)

BIO17 = Precipitation of Driest Quarter (mm)

BIO18 = Precipitation of Warmest Quarter (mm)

BIO19 = Precipitation of Coldest Quarter (mm)

## Appendix B

**Table A1:** The table presents the loadings of 19 BIO factors on principal components 1–5.

	PC1	PC2	PC3	PC4	PC5
BIO1	0.2429	−0.3024	0.0071	0.0418	0.0484
BIO2	−0.2506	−0.1029	−0.0839	0.2398	−0.5201
BIO3	−0.0071	−0.0858	−0.5265	0.4997	−0.3949
BIO4	−0.2505	−0.0185	0.3558	−0.1360	−0.2059
BIO5	0.0996	−0.4549	0.1793	−0.0598	−0.2647
BIO6	0.2761	−0.1548	−0.1609	0.0230	0.1440
BIO7	−0.2640	−0.0577	0.2762	−0.0571	−0.3009
BIO8	0.1153	−0.4252	0.2569	0.0237	0.0042
BIO9	0.2662	−0.1873	−0.1632	−0.0064	0.0383
BIO10	0.1573	−0.4088	0.2197	−0.0377	−0.0766
BIO11	0.2685	−0.1936	−0.1544	0.0797	0.1060
BIO12	0.2674	0.1868	0.1548	0.1058	−0.1290
BIO13	0.2165	0.1816	0.3010	0.3710	0.0069
BIO14	0.2612	0.1546	−0.0310	−0.2403	−0.3169
BIO15	−0.2352	−0.1290	0.1136	0.3439	0.1224
BIO16	0.2277	0.1957	0.2759	0.3224	−0.0276
BIO17	0.2653	0.1562	−0.0256	−0.2210	−0.2962
BIO18	0.2208	0.1984	0.2892	0.3350	−0.0127
BIO19	0.2561	0.1585	−0.0343	−0.2587	−0.3287

## Appendix C

**Table A2:** The table presents the proportion and cumulative proportion of variance explained by principal components 1 to 19 (PC1–PC19).

PC	Eigen Value	Proportion of Variance (%)	Cumulative Proportion (%)
1	11.0439	58.1257	58.12567
2	3.7064	19.5074	77.63305
3	1.9811	10.4269	88.05994
4	1.2678	6.6726	94.73251
5	0.6494	3.4178	98.15035
6	0.1902	1.0010	99.15132
7	0.0782	0.4118	99.56316
8	0.0317	0.1667	99.72984
9	0.0148	0.0781	99.80793
10	0.0097	0.0512	99.85909
11	0.0081	0.0427	99.90175
12	0.0068	0.0359	99.93762
13	0.0064	0.0337	99.97132
14	0.0020	0.0107	99.98203
15	0.0015	0.0081	99.99013
16	0.0011	0.0057	99.9958
17	0.0007	0.0034	99.99923
18	0.0001	0.0008	100
19	0.0000	0.0000	100
